# Outcome correlation of smear-positivity but culture-negativity during standard anti-tuberculosis treatment in Taiwan

**DOI:** 10.1186/s12879-015-0795-1

**Published:** 2015-02-18

**Authors:** Wen-Cheng Chao, Yi-Wen Huang, Ming-Chih Yu, Wen-Ta Yang, Chou-Jui Lin, Jen-Jyh Lee, Ruay-Ming Huang, Chi-Chang Shieh, Shun-Tien Chien, Jung-Yien Chien

**Affiliations:** Institute of Clinical Medicine, National Cheng Kung University Medical College, Tainan, Taiwan; Department of Internal Medicine, Taichung Veteran General Hospital Chiayi Branch, Chiayi, Taiwan; Chang-Hua Hospital, Ministry of Health and Welfare, Changhua, Taiwan; Department of Internal Medicine, Taipei Medical University-Wan Fang Hospital, Taipei, Taiwan; Taichung Hospital, Ministry of Health and Welfare, Taichung, Taiwan; Tao-Yuan Hospital, Ministry of Health and Welfare, Tao-Yuan, Taiwan; Department of Internal Medicine, Buddhist Tzu Chi General Hospital, Tzu Chi University, Hualien, Taiwan; Hua-Lien Hospital, Ministry of Health and Welfare, Hualien, Taiwan; Chest Hospital, Ministry of Health and Welfare, #864, Zhongshan Rd, Rende District, Tainan, 717 Taiwan; Graduate Institute of Clinical Medicine, National Taiwan University College of Medicine, Taipei, Taiwan

**Keywords:** Tuberculosis, Treatment, Severity, Cavity, Relapse

## Abstract

**Background:**

The appearance of smear-positivity but culture-negativity (SPCN) for acid-fast bacilli among sputum specimen is frequently found in pulmonary tuberculosis (TB) patients during treatment. This study aimed to investigate clinical risk factors, impacts on treatment course, and relapse pattern associated with sputum SPCN.

**Methods:**

We retrospectively enrolled 800 patients with culture-proven pulmonary TB who were receiving standard treatment and follow-up at six TB-referral hospitals in Taiwan between January 2006 and December 2007. Relevant patient characteristics and chemotherapy data were analyzed for associations with incidence of SPCN. Data from patients who relapsed within 3 years after completing treatment were analyzed for associations with SPCN during treatment.

**Results:**

Of the 800 subjects, 111 (13.8%) had sputum SPCN during treatment. Three factors were found to predict the development of SPCN; namely, high initial acid-fast staining grading (OR, 3.407; 95% CI, 2.090–5.553), cavitation on chest-X ray films (OR, 2.217; 95% CI, 1.359–3.615), and smoking (OR, 1.609; 95% CI, 1.006–2.841). Patients with SPCN had longer treatment duration (rifampicin: 284 ± 91 vs. 235 ± 69 days, *P* <0.001; isoniazid: 289 ± 90 vs. 234 ± 69 days, *P* < 0.001) than those without SPCN. Finally, the rate of relapse within 3 years of completing treatment was similar for groups with/without SPCN (2.7%, 3/111 vs. 1.0%, 7/689, respectively; *P* = 0.15).

**Conclusions:**

In conclusion, severity of infection was a major risk factor for SPCN during treatment; however, the relapse rate within 3 years of completing treatment was not affected by the appearance of SPCN.

## Background

Tuberculosis (TB) remains a leading health problem worldwide. The causal pathogen *Mycobacterium tuberculosis* (*Mtb*) has extraordinarily adaptive strategies for different stressful circumstances, including anti-mycobacterial treatment [1,2]. Because smear-positivity for acid-fast bacilli in sputum specimens indicates a high mycobacterial load, culture-positivity for mycobacteria is expected in most cases. However, during antimycobacterial treatment, smear-positivity for acid-fast bacilli may not equate with culture-positivity for mycobacteria. Two recent studies have reported culture-negativity with smear-positivity at the end of the second month of intensive treatment in 44.2% of patients (50/113) in Taiwan and 46.45% (40/86) in Cameroon [3,4]. Additionally, in our previous study investigating 111 patients with smear-positivity in the fifth month of treatment, we found that 71.6% of cases (83/111) had smear-positive but culture-negative (SPCN) sputum [5]. Given that treatment failure is currently defined as positive sputum smear or culture in the fifth month or later of treatment, it is potentially risky to assess treatment failure by sputum smear alone because of the possibility of SPCN [6]. Such SPCN phenomena have great clinical impact in the long period of waiting for culture results of smear-positive sputum samples. Uncertainty about culture results may lead to prolonged isolation and suspicion of treatment failure [7,8]. Therefore, longitudinal studies are required to investigate the outcomes of SPCN phenomenon during treatment. In this study, we aimed to investigate risk factors, including severity of TB and clinical predisposing factors for TB, and their impacts on treatment and relapse of patients with sputum SPCN.

## Methods

This study was conducted retrospectively in six TB-referral hospitals in Taiwan. The Directly Observed Therapy (DOT) program was launched in Taiwan in April 2006 and DOT coverage of smear positive cases reached 92.6% in 2007 [9].

**Figure 1:**
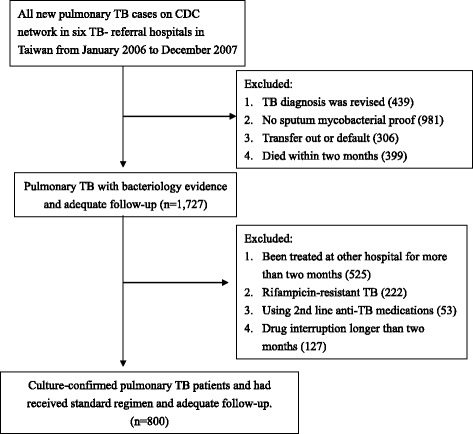
**Flow chart showing enrolment process.**

All patients reported as new pulmonary TB cases from January 2006 to December 2007 were identified by reviewing the databases of the Central Disease Control (CDC), Taiwan. From January 2006 to December 2007, 3852 patients were identified as new cases in the six TB referral hospitals. Patients were excluded if the TB diagnosis had been revised (439), there was no mycobacterial evidence of TB (981), or they had an inadequate duration of follow-up resulting from being transferred out (306) or dying within the initial 2 months (399). Patients who had received initial phase treatment at other hospitals (525), had rifampicin-resistant TB (222), were unable to tolerate first line treatment (53), or whose medication had been interrupted for longer than 2 months (127) were also excluded. Thus, 800 patients with culture-proven TB who had received the standard regimen and undergone adequate follow-up were enrolled in this study. The process of enrollment is summarized in Figure 1.

Cases of relapse were also identified by reviewing the databases of the CDC; and diagnoses of relapse were based on both chest-X ray films (CXR) and adequate sputum mycobacterial evidence. Sputum SPCN was defined as sputum that was smear-positive for acid-fast bacilli but did not yield any mycobacteria on either liquid or solid media. Our research was performed in compliance with the Helsinki Declaration and approval was obtained from our local ethics committee (Institutional Review Board of the Chung Shan Medical University Hospital, Reference No: CS11051).

In 2004, Taiwan CDC established the National Reference Laboratory of Mycobacteriology, which is responsible for formulating standard operating procedures and assessing and regulating the quality control programs of all laboratories in the mycobacterial laboratory examination network [10]. Briefly, the relevant standard procedures were performed as follows. Early-morning sputum samples were decontaminated with NaOH. Ziehl–Nielsen stained smears were examined and graded according to the American Thoracic Society guidelines [11]. As for mycobacterial culture, solid culture media, such as Lowenstein–Jensen media and 7H10 or 7H11 media, and liquid culture media, such as BacT/ALERT 3D (bioMerieux, Lyon, France) and the BACTEC MGIT (BACTEC Mycobacterium Growth Indicator Tube, MGIT 960 system, Becton-Dickinson, Sparks, MD, USA) were used. For identification, in addition to morphology and growth rate of the bacterial colonies, biochemical tests, and molecular assays were used to identify *Mtb* [12]. In accordance with the tuberculosis treatment guidelines in Taiwan [13], all patients’ sputum smears and cultures had been checked in the second and fifth months after initiation of treatment; monthly sputum checks had also been performed until sputum conversion in all patients with positive smears.

The medical records of enrolled patients were reviewed to obtain data on associated medical conditions, laboratory findings, hemoglobin A_1_C at the start of anti-TB treatment, serial sputum results, treatment regimen, and outcomes. CXRs at the start of treatment were evaluated by two pulmonologists from the Chest Hospital who were blinded to the clinical data. When their interpretations differed, the image was further reviewed by a third chest specialist who was also blinded to the results.

### Statistics

Data are presented as the frequencies (n) or percentages (%) for categorical factors and as means ± standard deviations for continuous factors. Categorical variables were compared using the χ^2^ test and differences in continuous variables were analyzed using the Mann–Whitney U test. Multivariate logistic regression analysis was used to determine independent variables that predicted SPCN. The Cochran–Armitage trend test was used to assess differences in trends in acid-fast smear (AFS) grading and cavity size in patients with or without sputum SPCN. Statistical significance was set at *P* < 0.05, two-sided. All data were analyzed using SPSS version 16.0 (SPSS, Chicago, IL, USA).

### Ethical approval

This study was approved by the Institutional Review Board of the Chung Shan Medical University Hospital (CSMUH No: CS11051).

## Results

Of the 800 enrolled cases, 111 (13.9%) developed SPCN in sputum during their treatment. Age, body weight, clinical symptoms, and laboratory findings were similar between patients with/without SPCN (Table 1). Patients with sputum SPCN were more likely to be male (79% vs. 69%, *P* = 0.033), to be smokers (57% vs. 36%, *P* < 0.001), to consume alcohol (32% vs. 20%, *P* = 0.008), and to have type 2 diabetes mellitus (34% vs. 21%, *P* = 0.003) than patients without sputum SPCN. Patients with sputum SPCN clearly had more severe TB than those without sputum SPCN, as evidenced by the former being more likely to have cavitated lesions (65% vs. 31%, *P* < 0.001) and large cavities (>4 cm) (42% vs. 14%, *P* < 0.001) on CXRs as well as higher sputum AFS grading than patients without sputum SPCN. On univariate analysis, the statistical power for cavitated lesions (*P* < 0.001), AFS grading (*P* < 0.001), and smoking (*P* < 0.001) were stronger than for maleness (*P* = 0.033), alcohol consumption (*P* = 0.008), and type 2 diabetes mellitus (*P* = 0.003), which may explain why subsequent multivariate analysis found that only cavitated lesions, AFS grading, and smoking predicted development of SPCN.

**Table 1:** **Characteristics of patients with and without sputum SPCN during treatment**

	**All**	**Sputum SPCN (+)**	**Sputum SPCN (−)**	**P value**
	**N = 800**	**N = 111**	**N = 689**	
**Basic data**				
Age (years)	55 ± 20	54 ± 18	55 ± 21	p = 0.784
Sex (male)	564 (71)	88 (79)	476 (69)	p = 0.033
Body Weight (kgs)	57 ± 11	56 ± 11	57 ± 11	p = 0.332
Smoking	311 (39)	63 (57)	248 (36)	p < 0.001
Alcohol consumption	171 (21)	35 (32)	136 (20)	p = 0.008
Diabetes mellitus	182 (23)	38 (34)	144 (21)	p = 0.003
HbA1C (%)	9.8 ± 3.2	10.6 ± 2.8	9.6 ± 3.3	p = 0.149
**Chest-X ray findings**				
Cavity formation	285 (36)	72 (65)	213 (31)	p < 0.001
Cavity > = 4 cm	146 (18)	47 (42)	99 (14)	p < 0.001
Pleural effusion	101 (13)	15 (14)	86 (12)	p = 0.759
**Sputum acid-fast stain**				p < 0.001
Negative	376 (47)	5 (5)	371 (54)	
1+	162 (20)	32 (29)	130 (19)	
2+	106 (13)	25 (23)	81 (12)	
3+	73 (9)	18 (16)	55 (8)	
4+	83 (10)	31 (28)	52 (8)	
**Symptoms**				
Cough	557 (70)	83 (75)	474 (69)	p = 0.354
Haemoptysis	74 (9)	12 (14)	62 (9)	p = 0.727
Fever	141 (18)	21 (19)	120 (17)	p = 0.893
Body weight loss (>5%)	194 (24)	32 (29)	162 (24)	p = 0.468
**Laboratory findings**				
WBC (cells/μl)	8469 ± 6840	8758 ± 2772	8423 ± 7287	p = 0.136
Hemoglobin (g/dL)	13 ± 2	13 ± 2	13 ± 2	p = 0.995
Albumin (mg/dL)	3.7 ± 2.2	3.4 ± 0.6	3.8 ± 2.5	p = 0.390
Creatinine (mg/dL)	1.1 ± 0.8	1.09 ± 0.5	1.15 ± 0.8	p = 0.450

Data are presented as N (%) or mean ± standard deviation.

*Abbreviations: SPCN* smear-positive but culture-negative, *HbA1C* Hemoglobin A1c.

**Figure 2:**
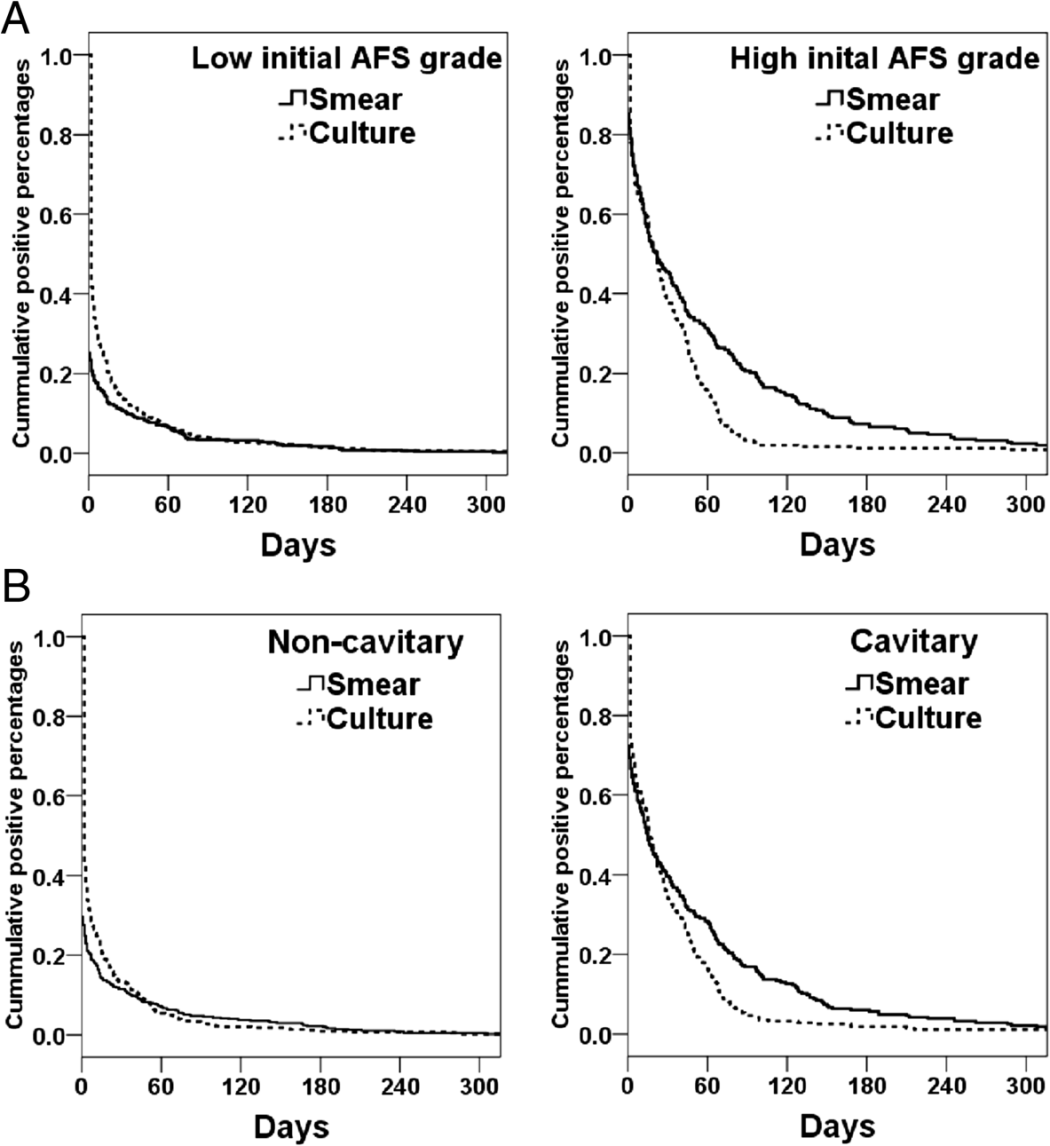
**Cumulative percentages of positive sputum mycobacterial smear (solid line) and positive sputum mycobacterial culture (dotted line) in patients with low (≤1+) and high acid-fast stain grading (>1+) at the start of treatment (A), and patients with and without cavity formation on chest-X ray films (B).** SPCN, smear-positive but culture-negative.

To clarify the time course of SPCN and the effect of TB severity on the development of SPCN, we correlated sputum smear and culture conversion curve with TB severity. In patients with low initial AFS grading or no cavities on CXRs, the sputum smear conversion and culture conversion curves were close to each other and sputum smear conversion mostly preceded sputum culture conversion (Figure 2). However, in patients with high initial AFS grading or cavities on CXR, there was discordance between sputum smear and culture conversion and culture conversion preceded sputum smear conversion.

**Figure 3:**
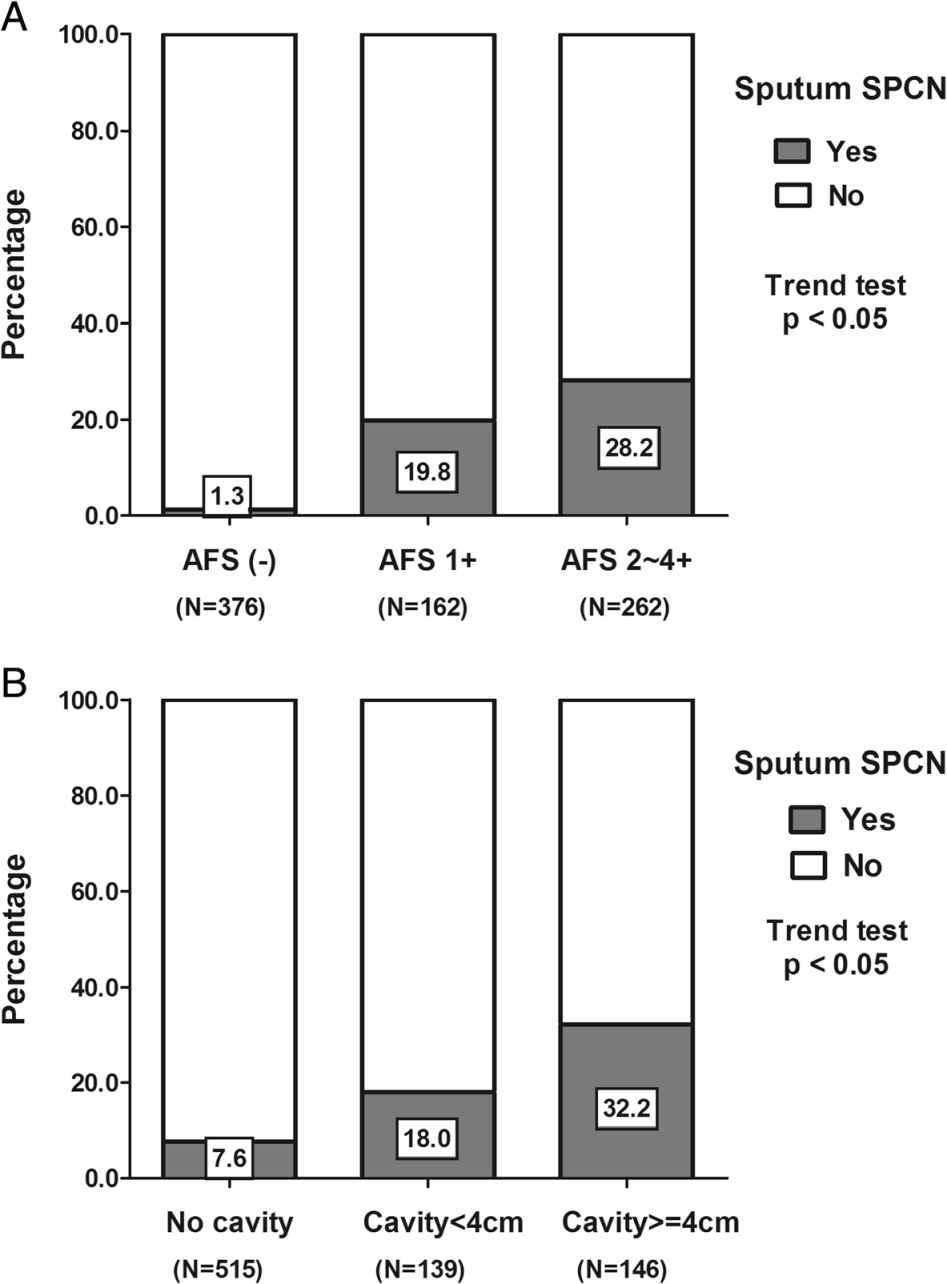
**Percentages of sputum SPCN in patients according to acid fast staining grade (A) and severity of cavitation (no cavities, cavities < 4 cm, and cavities > 4 cm) on chest-X ray films (B).**

Multivariate logistical regression identified three variables that were independently associated with the presence of sputum SPCN during treatment (Table 2). These were high initial acid-fast stain grading (>1+) (OR, 3.407; 95% CI, 2.090–5.553), cavities on CXR (OR, 2.217; 95% CI, 1.359–3.615), and smoking (OR, 1.609; 95% CI, 1.006–2.841). To further explore the effect of TB severity on the development of sputum SPCN, we subdivided patients with cavities on CXR into those with cavities larger or less than 4 cm in diameter and their AFS grading was classified as negative, 1+ and ≥2+. We found that proportions of sputum SPCN increased with TB severity as assessed by both cavitation and AFS grading in a dose-dependent manner (Figure 3). Thus, taken together, the data demonstrated that severe infection, characterized by high initial sputum AFS grading and cavities on CXR, was the key risk factor for the occurrence of sputum SPCN during anti-tuberculous treatment.

**Table 2:** **Multivariate logistic regression for sputum SPCN in TB patients**

**Characteristics**	**Multivariate**	
	***p*** **value**	**OR (95% C.I.)**
Age, per 1 year increment	0.826	
Gender, male vs. female	0.453	
Cavitation on chest X ray	0.001	2.217 (1.359 - 3.615)
High initial acid fast stain grading*	<0.001	3.407 (2.090 - 5.553)
Smoking	0.047	1.690 (1.006 - 2.841)
Alcohol consumption	0.541	
Diabetes mellitus	0.319	
WBC (per 1 cell/dLdecrement)	0.590	
Hemoglobin, (per 1 g/dLdecrement)	0.577	

* > 1+; SPCN, smear positive but culture negative; OR: Odds ration; C.I.: Confidence interval; WBC: White blood count.

To further investigate the clinical impact of sputum SPCN, we assessed treatment regimens, outcomes, and relapse rate during the 3-year follow-up after completing treatment (Table 3) and found that the 111 patients with sputum SPCN received treatment for longer than the 689 without sputum SPCN. The durations of treatment with rifampicin and isoniazid, the two most important medications in the continuation phase, were both longer in patients with than without sputum SPCN (rifampicin: 284 ± 91 vs. 235 ± 69 days, respectively, *P* < 0.001; isoniazid: 289 ± 90 vs. 234 ± 69 days, respectively, *P* < 0.001). Other first line medications used in the initial phase, including ethambutol, pyrazinamide, and streptomycin, were also administered for longer in patients with sputum SPCN than in those without sputum SPCN, although the difference was not as significant as for rifampicin and isoniazid. A small proportion of patients received fluoroquinolones because of adverse effects to first line medications; there was no difference in use of fluoroquinolones between two groups. Interestingly, we found that patients with sputum SPCN received treatment for longer after the last positive culture than did those without sputum SPCN (261 ± 72 vs. 220 ± 73, respectively; *P* < 0.001).

**Table 3:** **Treatment and outcome of patients with and without sputum SPCN**

	**SPCN (+)**	**SPCN (−)**	**P value**
	**N = 111**	**N = 689**	
**Treatment regimen**			
Isoniazide (days)	289 ± 90	234 ± 71	p < 0.001
Rifampicin (days)	284 ± 91	235 ± 69	p < 0.001
Ethambutol (days)	228 ± 105	191 ± 96	p = 0.001
Pyrazinamide (days)	137 ± 136	90 ± 74	p = 0.001
Streptomycin (days) (total cases)	84 ± 76 (18)	43 ± 34 (39)	p = 0.036
Levofloxacin (days) (total cases)	132 ± 141 (11)	172 ± 196 (17)	p = 0.559
Moxifloxacin (days) (total cases)	215 ± 120 (6)	162 ± 146 (13)	p = 0.456
**Treatment days after final positive culture**	261 ± 72	220 ± 73	p < 0.001
**DOT**	92 (83)	475 (69)	p = 0.001
**Complete treatment**	109 (98)	679 (99)	p = 0.677
**Sputum smear and culture status during treatment**			
**After treatment for 2 months**			
Smear positive	84 (76)	32 (5)	p < 0.001*
Culture positive	22 (20)	50 (7)	p < 0.001*
**After treatment for 5 months**			
Smear positive	26 (23)	9 (1)	p < 0.001*
Culture positive	1 (1)	14 (2)	p = 0.707*
**After treatment for 9 months**			
Smear positive	6 (5)	4 (1)	p = 0.001*
Culture positive	0 (0)	6 (1)	p = 0.407*
**Relapse within 3 years**	3 (3)	7 (1)	p = 0.150*

Data are presented as N (%) or mean ± standard deviation; SPCN, smear-positive but culture-negative; DOT, directly observed therapy.

*Fisher’s exact test.

The percentage of patients with culture positivity was similarly low between the two groups after treatment for 5 months (0.9%, 1/111 vs. 2.0%, 14/689; *P* = 0.707); however, even after treatment for 9 months the percentage of patients with smear positivity remained higher in patients with sputum SPCN than in those without it (5.4%, 6/111 vs. 0.6%, 4/689, respectively; *P* = 0.001). Finally, although the 111 patients with sputum SPCN received treatment for longer and had slower smear conversion than the 689 patients without sputum SPCN, there were no differences between these two groups in terms of completed treatment rate (98% vs. 99%, respectively, *P* = 0.677) and relapse rate after follow-up for 3 years (3% vs. 1%, respectively, *P* = 0.150). The data regarding relapsed cases are summarized in Table 4.

**Table 4:** **Characteristics of the 10 relapse cases** ^**a**^

**No**	**Sputum SPCN**	**Co-morbidity**	**CXR (cavity)**	**DOT**	**Treatment regimen**
1	No	Nil	NO	Yes	HER (194 d), Z (8 d)
2	No	Nil	NO	No	HER (184 d), Z (63 d)
3	No	Nil	NO	Yes	HER (182 d), Z (77 d)
4	No	Alcoholism	Yes	Yes	HER (328 d), Z (17 d)
5	Yes	DM	No	Yes	HER (252 d), Z (84 d)
6^#^	No	DM	Yes	Yes	HR (539 d), EZ (32 d)
7	No	DM	Yes	No	HER (310d), Z (30 d)
8	Yes	Alcoholism	No	Yes	HER (358 d), Z (12 d)
9	Yes	DM/Alcoholism	Yes	Yes	HER (343 d)
10	No	Nil	Yes	Yes	HR (231 d), E (21 d), Z (15 d)

^#^MDR-TB while relapse; SPCN (smear positive but culture negative); DM, diabetes mellitus; DOT, directly observed therapy; H, Isoniazide; E, Ethambutol; R, Rifampicin; Z, Pyrazinamide.

^a^Patients who died during the study were excluded.

Taken together, our data show that sputum culture conversion and relapse rates are not affected by the sputum SPCN, possibly because patients with SPCN received treatment for longer than those without sputum SPCN.

## Discussion

This study aimed to investigate the risk factors and clinical impact of the phenomenon of sputum SPCN. We found that severe pulmonary TB, as evidenced both by cavitation formation on CXR and high sputum AFS grading, and smoking contributed to the occurrence of sputum SPCN during treatment. Patients with sputum SPCN received continuation phase treatment for longer than patients without sputum SPCN; however, the relapse rate within 3 years after completion of treatment was similar between the two groups.

Our data showed cavitation on CXR is an important risk factor for sputum SPCN. Cavitation formation, a well-known characteristic presentation of TB infection, is believed to result from caseous granulomatous inflammation. With advances in knowledge regarding TB immunity, granuloma formation has been subdivided into highly-structured solid granulomas that contain *Mtb* and caseous granulomas with less-organized structures and liquefied centers, which lead to cavity formation [14,15]. Therefore, the strong correlation between cavitation and the occurrence of SPCN mycobacteria indicates that caseous granulomas, which contain numerous *Mtb* released from dead macrophages, may be the major source of SPCN mycobacteria. We therefore surmised that sputum SPCN may be a clinical indicator of host-pathogen interactions in the lungs during TB infection.

SPCN acid-fast bacilli were previously considered to be dead bacilli; however, accumulating evidence has now shown that culturability does not equal viability. Rather, SPCN bacilli can be viable but non-culturable (VBNC) and culturability can be restored with additional nutrient factors, including mycolic acid or proteins called resuscitation-promoting factors [16,17]. Recently, Mukamolova *et al.* used culture medium with and without resuscitation-promoting factors and demonstrated not only the presence of VBNC *Mtb* but also an increased proportion of VBNC *Mtb* during treatment of pulmonary TB infection [18]. These findings provide evidence of an adaptive phenotypic switch of mycobacteria after exposure to anti-TB treatment; such phenotype switches during treatment may lead to drug resistance and relapse [19]. In this study, we found that patients with sputum SPCN received treatment for longer after the final positive culture than patients without sputum SPCN (261 ± 72 vs. 220 ± 73, respectively; *P* < 0.001) (Table 3), the duration of treatment having been decided by the TB committees in each TB referral center. When faced with sputum smear-positivity near the end of the scheduled treatment course without other evidence of treatment failure, members of these committees tended to suggest continuation of treatment and follow-up of culture results rather than immediate modification of the drug regimen. The final treatment outcome and relapse rate were similar between the two groups. Importantly, the three patients who relapsed with sputum SPCN during initial treatment did not develop any new drug resistance. Therefore, our data provide evidence that continuation of treatment may be an acceptable and practical strategy for treating patients with sputum SPCN. However, more studies are needed to establish the best treatment regimen for these patients.

The long turnaround time for culture results creates several clinical dilemmas when treating patients with severe TB and sputum SPCN. Persistence of positive AFS in sputum often leads to prolonged isolation and suspicion of treatment failure [7,8]. Our data show that sputum SPCN mostly develops 157 ± 95 days after the start of treatment. Specifically, at the end of the second month, 14.5% of all patients (116/800) were smear-positive, but only 46.6% (54/116) of these smear-positive patients were culture-positive. Similarly, at the end of the fifth month, 4.4% of all patients (35/800) were smear-positive, but only 28.6% (10/35) of these smear-positive patients were culture-positive. Our data are fully consistent with those of another study conducted in Taiwan that reported 44.2% culture positivity (50/113) in 113 patients who were smear-positive at the end of the second month [3]. The data at the end of the fifth month are also consistent with those of our previous study, which showed only 25.2% (28/113) culture positivity in patients who were smear-positive at the end of the fifth month [5]. This longitudinal study showed that SPCN develops as early as 30 days after initiating treatment and is rarely found after 240 days of treatment (Figure 2). Therefore, AFS alone should not be used as the sole means of assessing treatment response during this period. Instead, as we showed in our previous study, multi-dimensional assessment, including culture conversion after initial phase treatment, CXR improvement, directly observed short-course therapy (DOTS), and AFS grading ≥3+ or not are crucial to thorough evaluation of treatment responses in patients with severe pulmonary TB [5].

Interestingly, we identified smoking as a minor risk factor for sputum SPCN in this study. Smoking, including passive smoking, has been found to be associated with active TB in Taiwan and other countries; the suppressive effect of smoking on airway defense has been assumed to be the causative factor [20-22]. Moreover, smoking was recently found to affect the rate of 2-month culture conversion during treatment [23]. However, whether the immune-suppressive effects of smoking also contribute to the development of sputum SPCN in pulmonary TB patients remains to be clarified.

Finally, SPCN is especially important in countries with limited laboratory capacity. In Taiwan, the laboratory capacity is adequate and all sputum samples are sent for both smear and culture. However, in many countries diagnosis of TB and assessment of treatment outcome still relies on sputum smear only. In these countries, the strong possibility of SPCN during anti-TB treatment in patients with severe TB infection should be taken into account when assessing treatment failure. As we showed in our previous study, multi-dimensional assessment, quality patient supervision, and chest radiography are crucial for avoiding unnecessary modification of treatment regimens [5].

This study has several noteworthy limitations. First, the viability of organisms in SPCN sputum cannot be ascertained under routine standard culture conditions without adding additional resuscitation factors. Second, the standard decontamination procedures in sputum processing can decrease culturability. We believe the differential error was not caused by such an inhibitory effect on culturability in this study because all cases had positive cultures for *Mtb* in sputum on enrollment and the inhibitory effect on culturability was the same for all sputum samples throughout the study period. Third, we totally excluded patients with non-tuberculous mycobacteria (NTM) in this study, and only AFS-positive organisms that grew no colonies on either liquid or solid media were classified as SPCN. Therefore, we postulate that we may have underestimated the true prevalence of sputum SPCN *Mtb* in this study, rather than amplified it, if there was any NTM contamination.

## Conclusions

This study provides clinical evidence of the previously ignored sputum SPCN phenomenon in patients with pulmonary TB receiving treatment. We have shown that severe TB infection, as evidenced by cavitation on CXR and high AFS grading in sputum, is the critical risk factor; smoking also contributes to the occurrence of sputum SPCN. Prolonging continuation phase treatment while awaiting results of culture of smear-positive samples appears to be a practical and safe strategy with an acceptable relapse rate; however, the ideal regimen for such patients has not yet been established.
